# Clinical profile and prevalence of odontogenic keratocysts in children and adolescents: A systematic review

**DOI:** 10.1016/j.jobcr.2026.101414

**Published:** 2026-02-09

**Authors:** Jéssica da Silva Cunha, Raisa Severino-Lazo, Joaquim Felipe Junior, Allan Vinícius Martins-de-Barros, Julliana Carvalho, Belmiro Cavalcanti do Egito Vasconcelos, Marianne Vasconcelos Carvalho

**Affiliations:** a University of Pernambuco, Oral HistoPathology Research Group, Postgraduate Program in Dentistry, Faculty of Dentistry of Pernambuco and Integrated Center of Pathological Anatomy, Oswaldo Cruz University Hospital, Recife, PE, 50040-200, Brazil; b University of Pernambuco, Department of Oral and Maxillofacial Surgery, Postgraduate Program in Dentistry, Faculty of Dentistry of Pernambuco, Recife, PE, 50040-200, Brazil; c University of Pernambuco, Postgraduate Program in Health Sciences, Faculty of Medical Sciences, Recife, PE, Brazil

**Keywords:** Recurrence, Jaw, Child, Adolescent

## Abstract

Odontogenic keratocyst (OKC) exhibits aggressive behavior and a high potential for recurrence. Despite being well-documented in adults, its characteristics in the pediatric population remain poorly understood. This systematic review aimed to analyze the clinical profile and treatment modalities of OKCs in children and adolescents, as well as their association with recurrence and follow-up duration. This systematic review was conducted following PRISMA guidelines and registered in PROSPERO (CRD42024612291). Risk of bias was assessed using the ROBINS-E tool, and statistical analysis was performed with the chi-square test. A total of 34 studies published between 2014 and 2024 were included, comprising 98 pediatric cases, both sporadic and syndromic. Both groups showed a slight male predominance, with a mean age of 12 years, and the most common symptom was extraoral swelling, with or without pain. The cases showed a predominance of lesions in the posterior mandible. Conservative approaches were the most frequently adopted treatment modality. The chi-square test revealed a statistically significant association between treatment modality and recurrence (p = 0.031), as well as between recurrence and follow-up duration (p = 0.005). Despite the variety of therapeutic strategies, recurrence was more strongly associated with follow-up duration than with the treatment approach itself. Based on these findings, we strongly recommend that all pediatric patients diagnosed with OKC undergo clinical and radiographic monitoring for a minimum of five years, regardless of the treatment performed, to ensure early detection and management of late recurrences.

## 1 Introduction

The odontogenic keratocyst (OKC) is a developmental cyst of odontogenic origin, first described by Philipsen in 1956.^1^ Due to its distinct biological behavior, the nature of this lesion has been a subject of long-standing debate. Reflecting this ambiguity, in 2005, the World Health Organization (WHO) reclassified OKC as a benign neoplasm—keratocystic odontogenic tumor (KCOT)—in the 3rd edition of its Classification of Head and Neck Tumors.^2^ However, in the 2017 edition, the lesion was once again categorized as an odontogenic cyst, based on growing evidence regarding its pathogenesis, molecular profile, and clinical characteristics.^3^^,^^4^

The PTCH1 mutation (9q22.3–q31) has been identified as a contributing factor to the aggressive biological behavior of odontogenic keratocysts (OKCs), including their high recurrence rate, through the constitutive activation of pathways that play a significant role in their pathogenesis.^5^, ^6^, ^7^ In addition, other genetic and molecular alterations have been investigated in OKCs, such as the BRAF p.V600E mutation; however, the available evidence remains limited and heterogeneous, with most studies reporting negative immunostaining or false-positive results, in contrast to the findings commonly observed in ameloblastomas.^8^, ^9^, ^10^, ^11^ Furthermore, recent studies have evaluated the expression of Secreted Cysteine-Rich Acidic Protein (SPARC) in OKCs, demonstrating its expression in fibroblasts within the cyst wall.^12^

OKCs can occur across a wide age range, with the highest incidence observed in the second and third decades of life, and a secondary peak reported in older adults. A slight male predominance has also been noted.^4^ In children and adolescents (aged 0–19 years), OKCs are less frequent, which has led to a scarcity of clinical data and limited information on long-term postoperative outcomes.^13^^,^^14^ Given its high recurrence rate and potential to compromise oral function and facial aesthetics, studies with extended follow-up periods are essential.^15^^,^^16^

The paucity of literature focused on pediatric patients requires surgeons to extrapolate data from studies on OKC in adults. This extrapolation may not always be appropriate due to the unique characteristics of the pediatric population, including the presence of permanent tooth buds and ongoing bone growth. Consequently, while more aggressive treatment options may result in fractures of the developing jaw, more conservative treatments are often chosen. However, this approach may carry a higher long-term recurrence risk, making postoperative follow-up crucial.^16^^,^^17^

It is essential to understand the prevalence and biological behavior of OKCs in children and adolescents. This topic is of great clinical and academic relevance, as further research can support evidence-based clinical decision-making and contribute to better outcomes and quality of life for pediatric patients.^18^ Therefore, this systematic review aims to provide a comprehensive analysis of the literature on this topic.

## 2 Materials and methods

### 2.1 Protocol and registration

This systematic review was conducted in accordance with the Preferred Reporting Items for Systematic Reviews and Meta-Analyses (PRISMA) guidelines,^19^ to identify, select, appraise, and synthesize relevant studies. The review protocol was registered in the International Prospective Register of Systematic Reviews (PROSPERO) under registration number CRD42024612291.

### 2.2 Research question

The research question guiding this review was: “What is the prevalence and clinical profile of odontogenic keratocysts in children and adolescents over the past ten years?” A PECOS strategy was applied, in which the population (P) comprised children and adolescents (0–19 years); Exposure (E) was a confirmed diagnosis of OKC; Comparison (C) was the absence of OKC; Outcomes (O) included demographic, clinical, and treatment-related characteristics, including conservative treatment, conservative treatment with adjuvant therapies, and radical treatment; and Study design (S) included observational studies.

### 2.3 Eligibility criteria

#### 2.3.1 Inclusion criteria

The inclusion criteria were: studies reporting a confirmed clinical and histopathological diagnosis of OKC according to WHO criteria; involving patients aged between 0 and 19 years; published since 2014; and reporting primary epidemiological data. The inclusion of studies published in the last ten years aimed to ensure the selection of the most recent evidence, providing an adequate time period to allow for meaningful discussion of trends and patterns relevant to the topic.

#### 2.3.2 Exclusion criteria

The exclusion criteria were: unavailability of the full text; uncertain or insufficiently confirmed diagnosis of OKC; studies based on animal models; and secondary studies (e.g., systematic, integrative, or exploratory reviews).

### 2.4 Information sources and search strategy

An extensive literature search was performed using the following electronic databases: MEDLINE/PubMed, Embase, Web of Science, Scopus, and Google Scholar. The search was limited to the previous ten years and was conducted without language restrictions. The search terms included: ((*pediatrics OR child OR children OR childhood OR infant OR adolescent OR teenager OR teen OR young OR juvenile OR “young people”) AND (odontogenic cysts OR keratocysts OR keratocysts odontogenic OR keratocyst odontogenic tumor))*. Additionally, the reference lists of all selected articles were manually screened to identify other potentially relevant studies.

### 2.5 Data extraction

Data from each included study: authorship, year of publication, country, number of patients, OKC classification (sporadic or syndromic), sex, mean age and age range, lesion site, clinical symptoms, treatment modality, recurrence, and follow-up duration.

### 2.6 Risk of bias

The risk of bias was assessed independently assessed by three reviewers (LNR, RJGSL, and MVC) using the ROBINS-E tool.^20^ This tool evaluates non-randomized studies of exposure across seven domains: bias due to confounding, bias in the measurement of exposure, bias in the selection of participants, bias due to post-exposure interventions, bias due to missing data, bias in the measurement of outcomes, and bias in the selection of reported results. Each domain was judged as having a risk of bias at one of four levels: low, some concerns, high, or very high. Any disagreements among reviewers were resolved through discussion until consensus was achieved.

### 2.7 Statistical analysis

Statistical analyses were performed using IBM SPSS Statistics (version 29.0). The Chi-square test (χ^2^) was used to assess associations between categorical variables, specifically between treatment modality and recurrence, and between recurrence and follow-up duration. Analyses were conducted separately for sporadic and syndromic OKC cases. A p-value of <0.05 was considered statistically significant. These analyses were exploratory in nature and were intended to assess associations rather than to generate pooled effect estimates.

### 2.8 Additional analysis

Inter-rater agreement for study inclusion was assessed using the Cohen's Kappa coefficient. Agreement was interpreted as follows: 0 (no agreement), <0.8 (moderate agreement), and ≥0.8 (near-perfect agreement). Disagreements were resolved through discussion to achieve consensus.

## 3 Results

### 3.1 Study selection

**Fig. 1:**
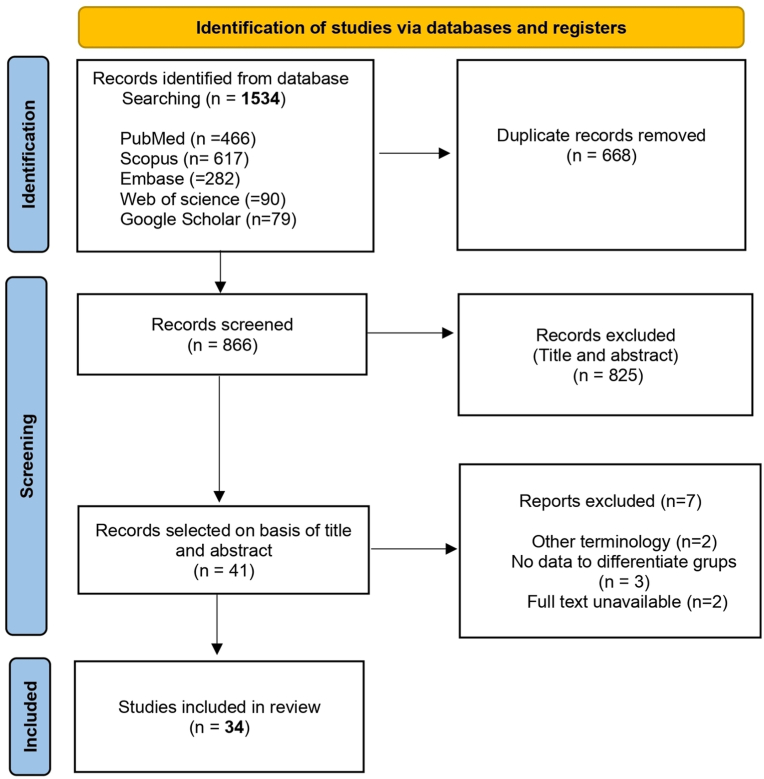
PRISMA flowchart of included studies.

The initial search of the databases identified 1534 articles: 466 in PubMed, 617 in Scopus, 282 in Embase, 90 in Web of Science, and 79 in Google Scholar. After excluding duplicate references using Rayyan software, titles and abstracts were analyzed. A total of 41 articles were selected for full-text analysis. Of these, seven articles were excluded: two used terminology not recommended by the WHO (‘peripheral OKC’) and described lesions that did not match the characteristics of OKC; three did not describe data by age group; and two were excluded due to unavailability of the full text. Consequently, this review encompasses 98 pediatric OKC cases reported in 34 articles. The study selection process is detailed in the PRISMA flowchart (Fig. 1). Inter-rater agreement for study inclusion, calculated using Cohen's Kappa coefficient, was almost perfect (Kappa = 0.90).

### 3.2 Study characteristics

**Fig. 2:**
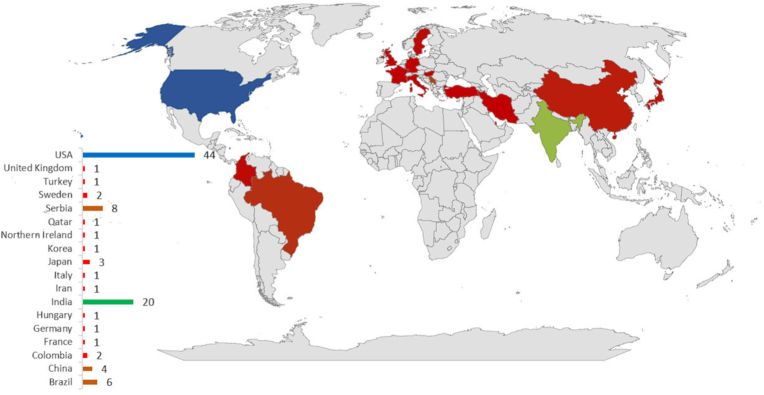
Distribution of pediatric OKC cases across included studies.

The 34 included articles were published between 2014 and 2024 and reported 98 pediatric OKC cases from 18 countries. The majority of cases were from the United States (n = 44) and India (n = 20), as shown in Fig. 2.

Cases were categorized into two groups: sporadic OKC (typically a single lesion not associated with a syndrome); and syndromic OKC (multiple cysts associated with Naevoid basal cell carcinoma syndrome - Gorlin syndrome, according to the classification system adopted by the original studies. This division aimed to highlight potential differences in demographic and clinical characteristics, treatment approaches, and recurrence patterns. Studies that included both types were divided, and cases were analyzed separately. Descriptive data for sporadic and syndromic cases are presented in Table 1, Table 2, respectively.

**Table 1:** Demographic and clinical characteristics of the sporadic OKC.

Author and Year of publication	Country	Sample	Demographics				Clinical			
			Mean Age (years)	Age range (years)	Sex	Anatomical location	Symptoms	Treatment	Rec.	Follow-up period (years)
Lacarbonara et al. 2014^21^	Italy	1	14	10–19	F	Posterior maxilla	Intraoral swelling	Conserv.	No	2
Urs et al. 2014^22^	India	11	16	10–19	6M/5F	Posterior mandible (6); anterior mandible (2)/posterior maxilla (1); anterior maxilla (2)	NR	Radic. (5) Conserv. (6)	NR	NR
Nomura et al. 2015^23^	Japan	1	17	10–19	F	Posterior maxilla	Nasal obstruction and pain	Decompress./marsupial.		1
Tkaczuk et al. 2015^24^	EUA	12	10.5	10–19	M/F	Mandible/maxilla	Extraoral swelling and pain	Conserv.	Yes	NR
Veerasamy et al. 2015^25^	India	1	15	10–19	M	Posterior maxilla	Toothache	Conserv.	No	2
Leung et al. 2016^26^	China	4	16.2	10–19	F	Posterior mandible	NR	Conserv. with adjuvant	Yes	16
					F	Posterior mandible				6.67
					M	Posterior maxilla				17.5
					M	Posterior mandible				10.42
Pejović et al. 2016^27^	Serbia	8	11.3	10–19	6M/2F	Posterior mandible (4); anterior mandible (1)/posterior maxilla (2); anterior maxilla (1)	Swelling	Conserv. with adjuvant	No	3.41
Rezende et al. 2016^28^	Brazil	1	14	10–14	F	Posterior mandible	Intra and extra oral swelling	Conserv.^a^	No	0.58
Alpy et al. 2017^29^	France	1	9	0–19	F	Posterior mandible	Intraoral swelling and suppurative fistula	Decompress./marsupial	No	7
Alstad et al. 2017^30^	Sweden	2	16.2	10–19	F	Posterior maxilla	Intraoral discharge	Conserv.	No	8
Guler et al. 2017^31^	Turkey	1	12	10–19	F	Posterior of bilateral mandible	Intraoral swelling	Decompress./marsupial	No	1.17
Peraza et al. 2017^32^	Colombia	2	17	10–19	M	Posterior mandible	Swelling	Conserv. with adjuvante^a^	No	8
					F					
Agrawal et al. 2018^33^	India	1	13	10–19	F	Posterior of bilateral mandible	Intra and extra oral swelling and pain	Conserv. With adjuvant	No	2
Morankar et al. 2018^34^	India	1	11	10–19	M	Posterior mandible	Intra and extra oral swelling	Decompress./marsupial	No	5
Harada et al. 2019^35^	Japan	1	8	0–9	F	Anterior and posterior maxilla and mandible	Intraoral swelling and pain	Conserv.^a^	Yes	10.67
Radia et al. 2019^36^	United kingdom	1	10	0–9	M	Anterior maxilla	No	Conserv. With adjuvant	No	0.5
Domingues et al. 2020^37^	Brazil	1	6	0–9	M	Posterior mandible	Loss of intraoral sensitivity	Decompress./marsupial	No	1
Pontes et al. 2021^38^	Brazil	1	0.75	0–9	F	Anterior maxilla	Extraoral swelling	Conserv.	No	5.42
Zhang et al. 2021^39^	EUA	1	14.5	10–19	9M/6F	Mandible (9)/maxilla (5)/both (1)	Swelling and pain	Conserv. With adjuvant	Yes	NR
Chida et al. 2023^40^	Japan	1	7	0–9	F	Posterior mandible	Intraoral swelling	Conserv.	No	0.5
Gupta et al. 2023^41^	India	1	14	10–19	M	Posterior mandible	Extra oral swelling	Conserv.^a^	No	5
Kumar et al. 2024^42^	India	1	13 y	10–19	M	Posterior mandible	Tooth misalignment	Conserv	NR	NR

M, male; F, female; NR, not reported; Rec, recurrence; Radic., radical; Conserv., conservative; Decompress/marsup, decompression/marsupialization; ^a^Decompress/marsup. before treatment.

**Table 2:** Demographic and clinical characteristics of the syndromic OKC.

Author and Year of publication	Country	Sample	Demographics				Clinical			
			Mean Age (years)	Age range (years)	Sex	Anatomical location	Symptoms	Treatment	Rec.	Follow-up period (years)
Anchlia et al. 2015^43^	India	1	18	10–19	M	Anterior maxilla; posterior of bilateral maxilla/posterior of bilateral mandible	Extra oral swelling	Conserv. with adjuvant	No	1
Anchlia et al. 2015^43^	India	1	18	10–19	M	Posterior of bilateral maxilla/posterior of bilateral mandible	No	Conserv. with adjuvant	No	1
Hajalioghli et al. 2015^44^	Iran	1	15	10–19	F	Posterior of bilateral maxilla/posterior of bilateral mandible	Parageusia and halitosis	No^b^	NR	NR
Pickrell et al. 2015^45^	EUA	11	12	10–19	F	Posterior of bilateral maxilla/posterior of bilateral mandible	Bilateral mandibular swelling	Conserv.	NR	NR
Ramanathan et al. 2015^46^	Qatar	1	9	0–19	F	Posterior of bilateral maxilla/posterior mandible	No	Conserv.	NR	NR
Tkaczuk et al. 2015^24^	EUA	7	10.5	10–19	M/F	Mandible/maxilla	Extraoral swelling and pain	Conserv.	Yes	NR
Mooney et al. 2016^47^	Northern Ireland	1	6	0–9	M	Posterior mandible	Extra oral swelling	Decompress./marsupial	No	1
										
Kim et al. 2017^48^	Korea	1	6	0–9	F	Posterior maxilla/posterior of bilateral mandible	Intraoral swelling	Decompress./marsupial	Yes	7
Santos et al. 2018^49^	Brazil	1	13	10–19	M	Posterior of bilateral maxilla/posterior of bilateral mandible	Extra oral swelling	Conserv.^a^	No	0.5
Lima et al. 2019^50^	Brazil	1	9	0–9	M	Anterior maxilla/posterior of bilateral mandible	Extra oral swelling	Conserv.^a^	NR	NR
Nilius et al. 2019^51^	Germany	1	9	0–9	M	Posterior maxilla/anterior mandible; posterior of bilateral mandible	Multiple tooth displacements	Conserv.	Yes	9
Gurdán et al. 2020^52^	Hungary	1	10	10–19	M	Posterior maxilla/posterior of bilateral mandible	No	Conserv.^a^	Yes	8
Zhang et al. 2021^39^	EUA	9	14.5	10–19	2M/7F	Mandible (1) maxilla (1)/Both (7)	Swelling and pain	Conser. with adjuvant	Yes	NR
Mondal et al. 2023^53^	India	1	13	10–19	M	Posterior maxilla/anterior mandible; posterior of bilateral mandible	Extra oral swelling and pain	Conserv.	NR	NR
Salahudheen et al. 2023^54^	India	1	17	10–19	F	Midline and anterior maxilla/posterior mandible	Extra oral swelling	Conserv. with adjuvant	No	5

M, male; F, female; NR, not reported; Rec, recurrence; Radic., radical; Conserv., conservative; Decompress/marsup, decompression/marsupialization; ^a^Decompress/marsup. before treatment; ^b^No There was no information on additional treatment.

### 3.3 Sporadic OKC

The sporadic group comprised 69 cases reported across 22 studies.^21^, ^22^, ^23^, ^24^, ^25^, ^26^, ^27^, ^28^, ^29^, ^30^, ^31^, ^32^, ^33^, ^34^, ^35^, ^36^, ^37^, ^38^, ^39^, ^40^, ^41^, ^42^ Among the 57 cases that reported sex, 27 (47.4%) were female and 30 (52.6%) were male. One study by Tkaczuk et al.^24^ reported 12 cases but did not specify the sex distribution.

The overall mean age was 12.06 years (SD: 3.83), with the youngest patient being 9 months old and the oldest 19 years.

Among the 69 cases, 57 provided clear information on anatomical location. Of these, 41 lesions (71.93%) were in the mandible and 16 (28.07%) in the maxilla. When the anatomical subsite was specified (n = 47), the posterior mandible was most frequently affected (n = 27; 57.45%), followed by the posterior maxilla (n = 10; 21.28%), anterior maxilla (n = 6; 12.76%), and anterior mandible (n = 4; 8.51%).

All 69 cases reported the general management approach. Broadly, 33 cases (47.83%) underwent conservative treatment, 31 cases (44.93%) received conservative treatment combined with adjuvant therapy, and 5 cases (7.24%) underwent radical treatment. However, specific procedural details were only provided in 23 studies.

Among the 23 studies that provided specific details on treatment modalities, five cases underwent marsupialization or decompression (decompression/marsupialization) as the sole therapeutic approach. Seven cases were treated conservatively through enucleation alone (conservative), without the use of adjuvant methods. Three cases followed a two-step conservative protocol (conservative∗), beginning with marsupialization or decompression followed by curettage. Six cases combined conservative treatment with adjuvant therapy (conservative with adjuvant), specifically curettage followed by application of Carnoy's solution. Finally, two cases employed a multistep approach that included prior decompression or marsupialization (conservative with adjuvant∗), followed by curettage and the use of Carnoy's solution.

**Fig. 3:**
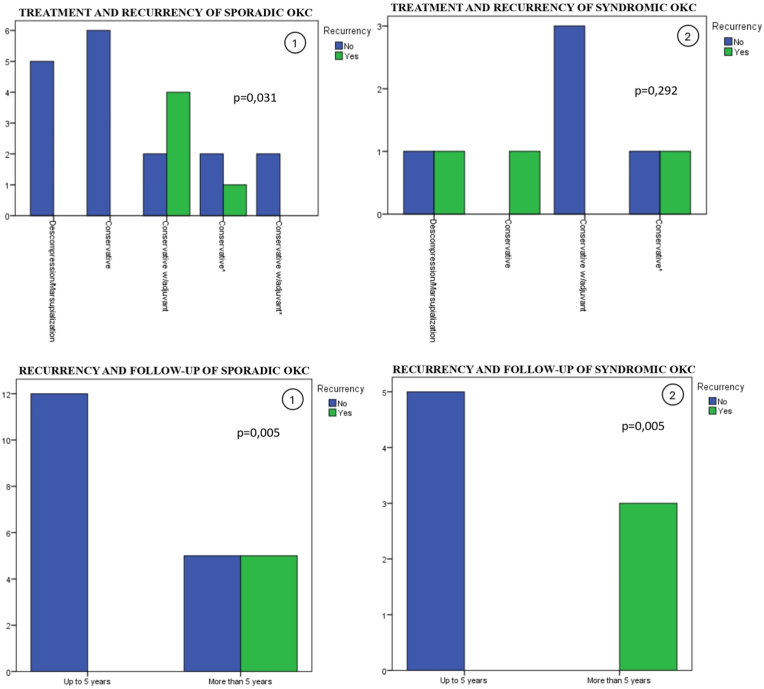
Statistical analysis panel of the association between treatment, recurrence, and follow-up of OKC.

Of these, only one case did not report whether recurrence occurred. Therefore, we performed an analysis based on the 22 cases that provided both treatment details and recurrence data. As shown in Fig. 3, recurrence was more frequently observed in cases treated with conservative approaches combined with adjuvant therapy (conservative with adjuvant), particularly when not preceded by decompression. In contrast, no recurrences were reported among cases treated solely with decompression/marsupialization or with conservative methods without adjuvants.

When analyzing the relationship between recurrence and follow-up duration, an important pattern emerged. As shown in Fig. 3, cases with a follow-up period longer than five years presented a significantly higher recurrence rate compared to those with follow-up periods of five years or less, regardless of the treatment modality applied. This association was statistically significant (p = 0.005).

### 3.4 Syndromic OKC

The syndromic OKC group comprised 22 cases.^43^, ^44^, ^45^, ^46^, ^47^, ^48^, ^49^, ^50^, ^51^, ^52^, ^53^, ^54^ Among these, 12 patients (54.55%) were female and 10 (45.45%) were male in studies that specified gender. As with the sporadic OKC group, the study by Tkaczuk et al.,^24^ which accounted for seven cases (31.82% of the total), did not provide information on the sex of the patients.

The mean age of patients with syndromic OKCs was 11.18 years (SD: 3.74), with an age range of 5–17 years. Among the 22 cases, 20 provided information on lesion location. The mandible was the most common site (n = 15; 75.0%), followed by the maxilla (n = 5; 25.0%). When the subsite was reported (n = 16), the posterior mandible was the most frequent (n = 11; 68.75%), followed by the posterior maxilla (n = 3; 18.75%) and anterior maxilla (n = 2; 12.5%). No syndromic OKC was located in the anterior mandible.

All 22 cases reported the general treatment approach. A total of 11 cases (50.0%) underwent conservative interventions or treatment, while 10 cases (45.45%) received conservative treatment combined with adjuvant therapy. Only one case (4.55%) was treated with a radical approach. However, only a subset of studies (n = 12) provided detailed descriptions of the treatment modalities used.

Among the 12 studies that provided specific details on treatment modalities, two (decompression/marsupialization) cases underwent marsupialization or decompression as the sole therapeutic approach. Four (conservative) cases were treated conservatively through enucleation alone, without the use of adjuvant methods. Three (conservative∗) cases followed a two-step conservative protocol, beginning with marsupialization or decompression followed by curettage. Three (conservative with adjuvant) cases combined conservative treatment with adjuvant therapy, specifically curettage followed by application of Carnoy's solution.

Of these, four cases did not report whether recurrence occurred. Therefore, we performed an analysis based on the 8 cases that provided both treatment details and recurrence data. As shown in Fig. 3, recurrence occurred across all treatment categories. Although recurrences appeared more frequent in cases managed without adjuvant therapy, the difference in recurrence rates among treatment groups was not statistically significant (p = 0.292).

As observed in the sporadic cases, the analysis of recurrence in relation to follow-up duration among syndromic OKCs revealed a similar pattern. Cases with a follow-up period longer than five years showed a higher recurrence rate compared to those followed for five years or less, regardless of the treatment approach.

### 3.5 Risk of bias

**Fig. 4:**
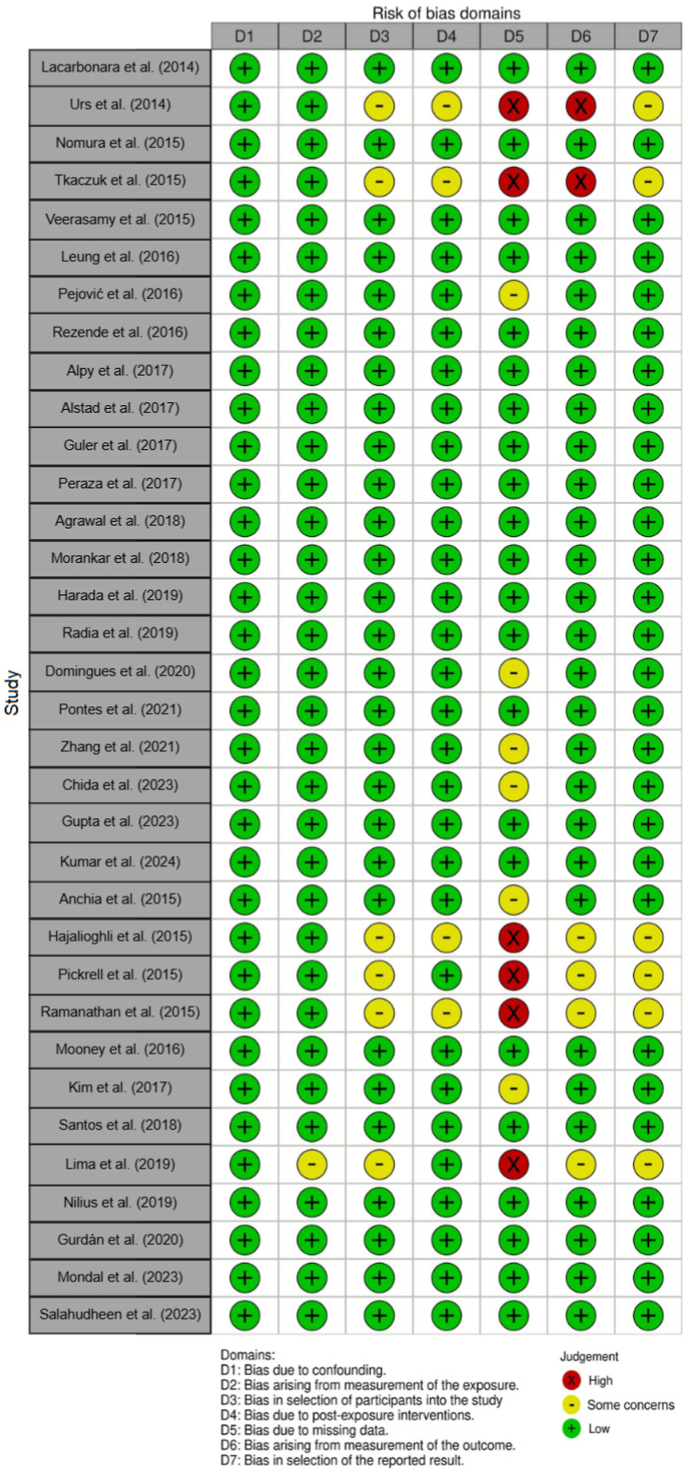
Risk of bias summary: reviewers' judgments for each checklist item across the non-randomized studies.

According to the ROBINS-E tool,^20^ the risk of bias in all included studies was classified as very high, high, some concerns, low, or no information. Most domains presented a low risk of bias, although some studies raised concerns regarding a high risk. In particular, the domain “bias due to missing data" was notable in the studies by Urs et al.^22^ and Tkaczuk et al.^24^ as they did not separately describe clinical information for 11 and 19 cases, respectively. Additionally, the study by Hajalioghli et al.^44^ did not specify whether a surgical procedure was performed following the maxillary biopsy. Finally, in the studies by Pickrell et al.^45^ Ramanathan et al.^46^ and Lima et al.^50^ essential data on recurrence and follow-up were not reported. The methodological quality and risk of bias of the selected studies were assessed according to the ROBINS-E^20^ checklist for observational studies. The results of the risk of bias assessment are presented in Fig. 4, with studies organized sequentially, including first sporadic OKC and, subsequently, syndromic OKC.

## 4 Discussion

Pediatric odontogenic keratocysts (OKCs) represent a unique diagnostic and therapeutic challenge due to their relatively low incidence, aggressive behavior, and potential for recurrence. While OKCs are well-documented in adults, literature addressing their occurrence in children and adolescents remains scarce. This gap hinders a precise understanding of their biological behavior and optimal management in younger individuals.^55^^,^^56^

This study provides a comprehensive synthesis of pediatric OKC cases from the past decade, combining epidemiological data with detailed clinical features and follow-up information. It adheres to the current WHO classification criteria^4^ and contributes to establishing clearer parameters for diagnosis and long-term surveillance in this age group.

Our systematic review identified 98 pediatric OKC cases across 18 countries, reflecting the lesion's global distribution. Similar to previous reports,^55^^,^^56^ a slight male predominance was observed in both sporadic and syndromic cases. The mean age at diagnosis was approximately 12 years in both groups, aligning with the peak incidence reported in the literature.^4^

Anatomically, OKCs demonstrated a clear predilection for the mandible, particularly the posterior region—a pattern consistent with other studies.^55^, ^56^, ^57^ In our sample, 71.93% of sporadic and 75% of syndromic OKCs occurred in the mandible. Notably, the posterior mandible was the most affected subsite in both groups. These findings support the WHO's statement that OKCs have a 4:1 mandibular-to-maxillary ratio and tend to present in the posterior mandible and ramus region.^4^

Symptoms were often absent or nonspecific, consistent with prior studies reporting that pediatric OKCs are frequently diagnosed incidentally.^56^^,^^58^ This underscores the importance of radiographic evaluation in asymptomatic children and adolescents, especially during orthodontic planning or assessing delayed tooth eruption.

The therapeutic approaches adopted in the included studies were predominantly conservative (47.83%), followed by conservative treatments with adjuvants (44.93%), and, less frequently, radical treatments (7.24%). However, only 23 of the 69 sporadic cases provided detailed descriptions of the sequence and specific components of the interventions. Among these, some studies reported exclusively the use of marsupialization or decompression without subsequent surgery.

It is important to emphasize that, although useful in certain pediatric contexts, these techniques are not considered definitive treatment. According to WHO,^4^^,^^59^ marsupialization and decompression are strategies frequently employed prior to enucleation or complete removal of the lesion, with the aim of facilitating surgery, reducing bone loss, and consequently lowering recurrence rates. The WHO also notes that the recurrence risk after traditional enucleation ranges from 20% to 30% but can be significantly reduced with adjunctive treatments such as curettage, peripheral ostectomy, cryotherapy, or chemical cauterization.

Adjuvant treatments are frequently used as adjuncts to both conservative and radical treatments, with Carnoy's solution being a prevalent chemical adjuvant. Although the efficacy of Carnoy's solution as an adjuvant agent is well established, concerns have been raised regarding its potential carcinogenicity. Consequently, new therapeutic interventions are being investigated, including modified Carnoy's solution, a chloroform-free compound, and 5-fluorouracil (5-FU), an antimetabolite that interferes with DNA synthesis in rapidly proliferating cells, leading to cellular apoptosis.^60^^,^^61^ Despite the absence of these new therapies in our research, they are emerging as effective tools, particularly in cases of lesions with high recurrence rates.

Recurrence is frequently associated with incomplete lesion removal and the presence of satellite cysts or epithelial remnants at the osseous margins.^35^ In this context, the adoption of a staged strategy—such as decompression followed by enucleation, with or without adjuvant use—is clinically coherent.

However, when comparing our findings with the existing literature, some apparent inconsistencies emerged. In the analysis of the 23 detailed cases, those treated solely with marsupialization or decompression showed no recurrence, while recurrences were more frequent among those managed with conservative interventions combined with adjuvants. Initially, these data could suggest a conclusion contrary to expectations, but upon examining the follow-up duration, a clear explanation emerged: the cases without recurrence had follow-up periods of less than five years, while recurrences occurred predominantly in cases with follow-up exceeding five years.

This finding was statistically significant in the group of sporadic OKCs (p = 0.005) and showed a similar trend in syndromic cases. Thus, our analysis reinforces that, more than the therapeutic modality itself, the duration of follow-up is critical for the detection of recurrence. Clinical and radiographic monitoring should be maintained for at least five years as an integral part of the treatment plan, especially in children and adolescents, whose bone and dental development may mask early signs of recurrence.^62^

Therefore, when discussing treatment modalities for pediatric OKCs, it is essential to recognize that interventions such as marsupialization and decompression are complementary and preparatory tools, and do not replace surgical removal of the lesion. The most effective treatment will always be one that combines an appropriate technical approach with prolonged and rigorous follow-up, ensuring not only immediate resolution of the lesion but also long-term recurrence prevention.^13^^,^^63^^,^^64^

Importantly, our findings highlight the need for long-term follow-up in pediatric patients, regardless of treatment modality. By identifying recurrence patterns in relation to treatment strategies and duration of follow-up, our results provide valuable evidence to guide clinical decision-making. Given the psychosocial and functional implications of maxillofacial surgery in children, optimizing management strategies based on age-specific data is of high clinical and public health relevance.^58^

### 4.1 Study limitations

This systematic review has several limitations. Many of the included studies provided incomplete clinical data, particularly regarding detailed descriptions of treatment, follow-up duration, and recurrence outcomes. Only a subset of studies offered sufficient information for more in-depth analysis. Furthermore, there was substantial methodological heterogeneity among the included articles, which hindered direct comparisons. The risk of bias assessment indicated that, although most domains showed low risk, notable weaknesses were identified, especially related to missing key data, such as recurrence outcomes and clarity regarding the interventions performed. These limitations highlight the need for future studies with greater methodological rigor, standardized reporting, and long-term follow-up, particularly in pediatric populations.

## 5 Conclusion

This systematic review indicates that odontogenic keratocysts (OKCs) in children and adolescents are more frequent in males, particularly around 12 years of age, a trend consistent with the available literature despite the limited number of studies. The results underscore the importance of clinical follow-up for at least five years, given the ongoing bone development in pediatric patients and the risk of recurrence. Recurrences were especially observed after longer follow-up periods, independent of treatment modality or syndromic association. Although conservative approaches remain the most suitable, the evidence reinforces the need for rigorous, long-term monitoring to detect late recurrences. These findings contribute to a deeper understanding of OKCs in children and adolescents and highlight the importance of evidence-based, age-specific follow-up protocols that address both prevalence patterns and long-term outcomes.

## Patient/guardian consent

As this research consisted of a general review of the existing literature, the consent of the patient/guardian was not required.

## Ethical statement

This general review used data from published studies and did not require ethical approval, as no new data was collected. Therefore, the Institution's Ethics Committee waived ethical approval.

## Source(s) of funding

This research has not received any specific grants from funding agencies in the public, commercial, or non-profit sectors.

## Declaration of competing interest

The authors declare that they have no known competing financial interests or personal relationships that could have appeared to influence the work reported in this paper.
